# Supplementary material for: Phage-assisted evolution of allosteric protein switches
Source: Nat Commun. 2026 Apr 14;17:3498. doi: 10.1038/s41467-026-71717-0 (PMC13079736; doi:10.1038/s41467-026-71717-0)
Supplement: Supplementary file 3 — Description of Additional Supplementary Files [file 41467_2026_71717_MOESM3_ESM.pdf]

Description of Additional Supplementary Files

File Name: Supplementary Data 1
Description: List of E Coli Strains, Circuits, SPs, mRFP Reporter Assay, and RAMPhaGE phages and plasmids

File Name: Supplementary Data 2
Description: List of Primer sequences

File Name: Supplementary Data 3
Description: Overview of PANCE Selection

File Name: Supplementary Data 4
Description: Overview of POGO-PANCE Selection

File Name: Supplementary Data 5
Description: Overview of Combined POGO-PANCE/RAMPhaGE Selection

File Name: Supplementary Data 6
Description: Defined Linker Library and Naming Convention
