# Supplementary material for: Phage-assisted evolution of allosteric protein switches
Source: Nat Commun. 2026 Apr 14;17:3498. doi: 10.1038/s41467-026-71717-0 (PMC13079736; doi:10.1038/s41467-026-71717-0)
Supplement: Supplementary file 5 — Reporting Summary [file 41467_2026_71717_MOESM5_ESM.pdf]

Corresponding author(s): Jan Mathony, Dominik Niopek

Last updated by author(s): Feb 25, 2026

Reporting Summary

Nature Portfolio wishes to improve the reproducibility of the work that we publish. This form provides structure for consistency and transparency in reporting. For further information on Nature Portfolio policies, see our Editorial Policies and the Editorial Policy Checklist.

Statistics

For all statistical analyses, confirm that the following items are present in the figure legend, table legend, main text, or Methods section.

n/a	Confirmed	
☐	☒	The exact sample size (*n*) for each experimental group/condition, given as a discrete number and unit of measurement
☐	☒	A statement on whether measurements were taken from distinct samples or whether the same sample was measured repeatedly
☒	☐	The statistical test(s) used AND whether they are one- or two-sided<br>*Only common tests should be described solely by name; describe more complex techniques in the Methods section.*
☒	☐	A description of all covariates tested
☒	☐	A description of any assumptions or corrections, such as tests of normality and adjustment for multiple comparisons
☐	☒	A full description of the statistical parameters including central tendency (e.g. means) or other basic estimates (e.g. regression coefficient) AND variation (e.g. standard deviation) or associated estimates of uncertainty (e.g. confidence intervals)
☒	☐	For null hypothesis testing, the test statistic (e.g. *F*, *t*, *r*) with confidence intervals, effect sizes, degrees of freedom and *P* value noted<br>*Give P values as exact values whenever suitable.*
☒	☐	For Bayesian analysis, information on the choice of priors and Markov chain Monte Carlo settings
☒	☐	For hierarchical and complex designs, identification of the appropriate level for tests and full reporting of outcomes
☐	☒	Estimates of effect sizes (e.g. Cohen's *d*, Pearson's *r*), indicating how they were calculated

Our web collection on statistics for biologists contains articles on many of the points above.

Software and code

Policy information about availability of computer code

Data collection	Fluorescence and optical density data were collected using a Tecan Infinite 200 Pro (TECAN) and analyzed via the i-control software (Tecan, version 2.0). Flow cytometry was performed on a Beckman Coulter CytoFLEX S cytometer instrument and data analyzed using the CytoFlow (v1.1.1) software. Nanopore sequencing was performed using the Oxford Nanopore Technologies (ONT) MinION platform with FLO-MIN114 flow cells and data was analyzed using the Oxford Nanopore Technologies' (ONT) Dorado basecaller (version 0.7.4+59ab908). Short-read sequencing was performed via the EZ-Amplicon sequencing service (Genewiz Azenta). Phage titer quantification by qPCR was performed on a Quantabio Q qPCR instrument (Quantabio) and analyzed using Q-qPCR Software v1.0.2.
Data analysis	Data analysis was performed in Python (v3.11.1) using the following libraries: pandas and numpy for data manipulation, scipy for statistical analyses, and matplotlib and seaborn for data visualization. Bioinformatic processing of sequencing data incorporated pysam and Biopython, alongside custom Python modules for DNA translation, variant calling, mutation enrichment analysis, and co-occurrence profiling. Flow cytometry data were analyzed using CytoFlow (v1.1.1), with Python (v3.6.7). Structural visualizations of AlphaFold3-predicted models were generated in PyMOL (v3.1.4.1). Plasmid maps were viewed and annotated using SnapGene (Dotmatics, v8.1). Bar plots were compiled and visualized using GraphPad Prism (v10). The secondary structure annotations in Fig. 3c were generated using a custom Python script, based on secondary structure predictions from PSIPRED (v4.0) via the PSIPRED Workbench webserver and domain annotations for AraC from InterPro. Figures were assembled in Adobe Illustrator (v6).<br>Scripts used for data analysis, RFP measurement data, as well as the tool for RAMPhaGE oligo pool design, are available on GitHub: https://github.com/Niopek-Lab/POGO_PANCE.git.

For manuscripts utilizing custom algorithms or software that are central to the research but not yet described in published literature, software must be made available to editors and reviewers. We strongly encourage code deposition in a community repository (e.g. GitHub). See the Nature Portfolio guidelines for submitting code & software for further information.

Data

Policy information about availability of data

All manuscripts must include a data availability statement. This statement should provide the following information, where applicable:

- Accession codes, unique identifiers, or web links for publicly available datasets
- A description of any restrictions on data availability
- For clinical datasets or third party data, please ensure that the statement adheres to our policy

Supplementary Tables 1-6 are available as Supplementary Data 1; Supplementary Table 1 contains all used strains and plasmid sequences. Raw measurement data is available as Supplementary Data 2. Relevant plasmids will be made available via Addgene. NGS raw data is available on Zenodo (doi:10.5281/zenodo.15650047).

Human research participants

Policy information about studies involving human research participants and Sex and Gender in Research.

Reporting on sex and gender	N/A
Population characteristics	N/A
Recruitment	N/A
Ethics oversight	N/A

Note that full information on the approval of the study protocol must also be provided in the manuscript.

Field-specific reporting

Please select the one below that is the best fit for your research. If you are not sure, read the appropriate sections before making your selection.

☒ Life sciences ☐ Behavioural & social sciences ☐ Ecological, evolutionary & environmental sciences

For a reference copy of the document with all sections, see nature.com/documents/nr-reporting-summary-flat.pdf

Life sciences study design

All studies must disclose on these points even when the disclosure is negative.

Sample size	No sample size calculation was performed. The sample size was determined based on pilot experiments and aligned to conventions in the field (Esvelt et al., Nature, 2011; Huang et al., Nat. Biotechnol., 2023; Mathony et al., Adv Sci. (Weinh), 2023; Fishman et al., Nat. Biotechnol., 2024)
Data exclusions	No experimental data were excluded from the analysis.
Replication	All attempts for replication were successful. The number of replicates performed is indicated in each figure legend, where applicable.
Randomization	No randomization was used, as samples and controls were treated side-by-side using the identical protocols and workflows for analysis. Also, the study did not involve human or animal subjects.
Blinding	No blinding was used, as the majority of data was automatically collected by machines and analyzed by standard workflows.

Reporting for specific materials, systems and methods

We require information from authors about some types of materials, experimental systems and methods used in many studies. Here, indicate whether each material, system or method listed is relevant to your study. If you are not sure if a list item applies to your research, read the appropriate section before selecting a response.

Materials & experimental systems

n/a	Involved in the study
☒	☐ Antibodies
☒	☐ Eukaryotic cell lines
☒	☐ Palaeontology and archaeology
☒	☐ Animals and other organisms
☒	☐ Clinical data
☒	☐ Dual use research of concern

Methods

n/a	Involved in the study
☒	☐ ChIP-seq
☐	☒ Flow cytometry
☒	☐ MRI-based neuroimaging

Flow Cytometry

Plots

Confirm that:

☒ The axis labels state the marker and fluorochrome used (e.g. CD4-FITC).

☒ The axis scales are clearly visible. Include numbers along axes only for bottom left plot of group (a 'group' is an analysis of identical markers).

☒ All plots are contour plots with outliers or pseudocolor plots.

☒ A numerical value for number of cells or percentage (with statistics) is provided.

Methodology

Sample preparation	Following 18 hours of culture growth, 2 µL of each sample were diluted into 200 µL of sterile 1x PBS (Roth, cat. No. 9150.1) in a 96-well flat-bottom plate (Greiner, polystyrene, cat. no. M2936) and analyzed using a CytoFLEX flow cytometer (Beckman Coulter, CytoFLEX S) operated at medium flow rate. For each sample, 20,000 events were recorded. Note that we do not show contour plots, but historgrams, since we only have a single channel (RFP).
Instrument	CytoFLEX flow cytometer (Beckman Coulter, CytoFLEX S)
Software	CytoFlow (v1.1.1)
Cell population abundance	N/A, since this is an RFP measurment from E. coli cultures.
Gating strategy	The gating strategy is shown in Supplementary Fig. 2

☒ Tick this box to confirm that a figure exemplifying the gating strategy is provided in the Supplementary Information.
