# Supplementary figures and images for: Phage-assisted evolution of allosteric protein switches
Source: Nat Commun. 2026 Apr 14;17:3498. doi: 10.1038/s41467-026-71717-0 (PMC13079736; doi:10.1038/s41467-026-71717-0)

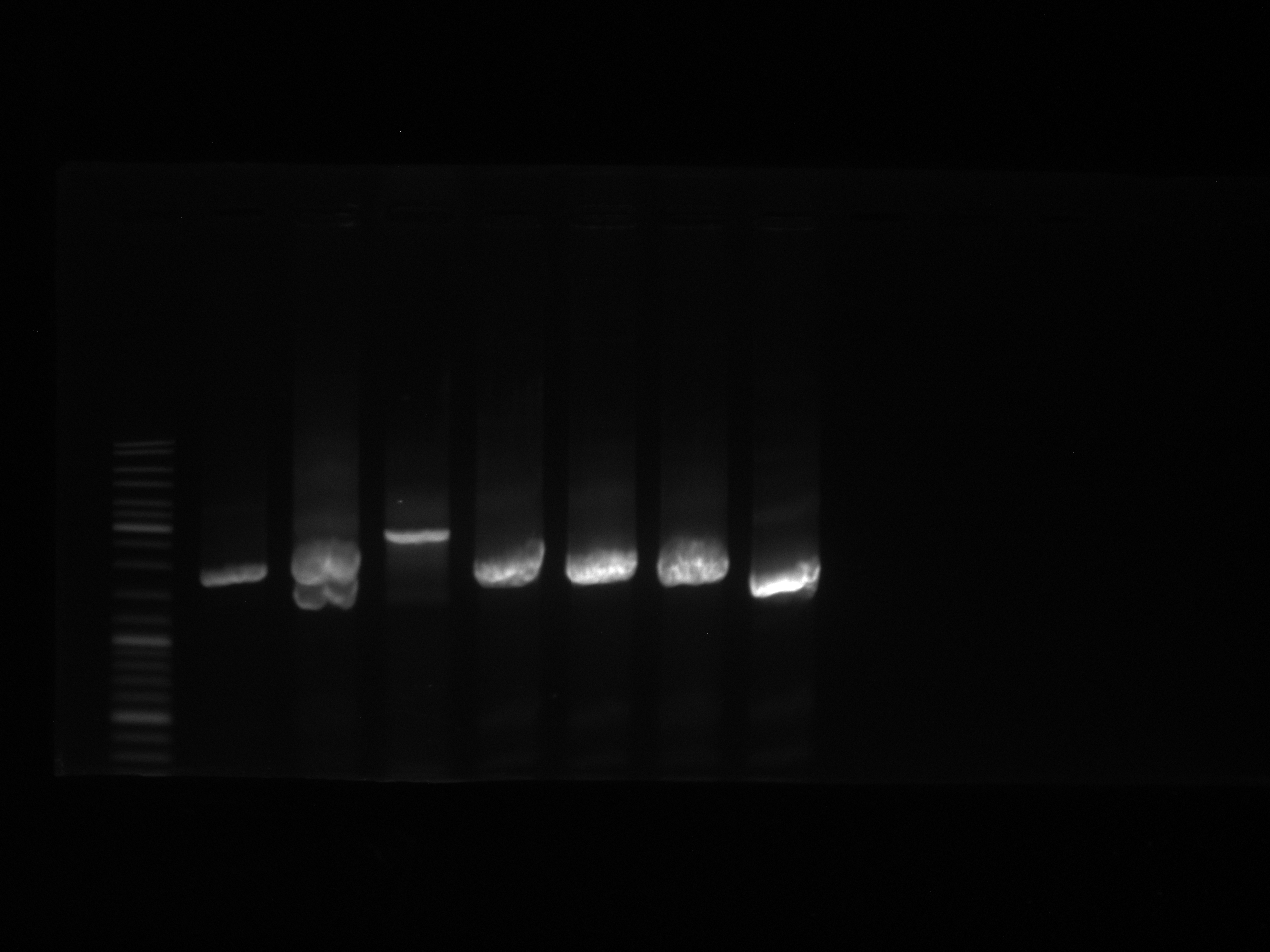

Supplement: Supplementary file 6 — Source Data [file 41467_2026_71717_MOESM6_ESM.zip › Source_Data/S19_b_Gel_L1-L3.tif]
